# Dopamine Transporter SPECT Imaging in Corticobasal Syndrome

**DOI:** 10.1371/journal.pone.0018301

**Published:** 2011-05-02

**Authors:** Roberto Cilia, Carlo Rossi, Daniela Frosini, Duccio Volterrani, Chiara Siri, Cristina Pagni, Riccardo Benti, Gianni Pezzoli, Ubaldo Bonuccelli, Angelo Antonini, Roberto Ceravolo

**Affiliations:** 1 Parkinson Institute, Istituti Clinici di Perfezionamento, Milan, Italy; 2 Department of Neurosciences, Section of Neurology, University of Pisa, Pisa, Italy; 3 Department of Nuclear Medicine, University of Pisa, Pisa, Italy; 4 Nuclear Medicine, IRCCS-Ospedale Maggiore, Milan, Italy; 5 Neurological Unit, Viareggio, Italy; 6 Institute of Neurology, IRCCS San Camillo, Venice, Italy; 7 University of Padua, Padua, Italy; Johns Hopkins, United States of America

## Abstract

**Objective:**

To investigate dopaminergic function in a large cohort of patients with corticobasal syndrome (CBS) and describe its relationship with clinical features in comparison to Parkinson's disease and healthy control subjects. In addition, we assessed prevalence and features of individuals with CBS and *in vivo* evidence of preserved nigral neuronal density.

**Background:**

Substantia nigra pars compacta (SNc) neuronal degeneration is a mandatory pathological criterion for definite corticobasal degeneration, though sporadic autopsy-proven cases with *ante-mortem* imaging evidence of preserved nigral terminals have been recently described.

**Methods:**

In this multicenter study, we investigated presynaptic nigrostriatal function in 36 outpatients fulfilling clinical criteria for “probable corticobasal degeneration” (age 71±7.3 years; disease duration 3.9±1.6 years), 37 PD and 24 healthy control subjects using FP-CIT single photon emission computed tomography. Clinical, neuropsychological, and magnetic resonance imaging assessment was performed to characterize CBS patients. Linear discriminant analysis was used to categorize normal vs. pathological scans.

**Results:**

FP-CIT binding reduction in patients with CBS was characterized by larger variability, more uniform reduction throughout the striatum and greater hemispheric asymmetry compared to PD. Moreover, there was no significant correlation between tracer uptake values and clinical features such as disease duration and severity. Despite all CBS subjects showed obvious bilateral extrapyramidal signs, FP-CIT uptake was found to be normal bilaterally in four CBS patients and only unilaterally in other four cases. Extensive clinical, neuropsychological and imaging assessment did not reveal remarkable differences between CBS subjects with normal vs. pathological FP-CIT uptake.

**Conclusions:**

Our findings support the hypothesis that extrapyramidal motor symptoms in CBS are not invariably associated with SNc neuronal degeneration and that supranigral factors may play a major role in several cases. CBS individuals with normal FP-CIT uptake do not show any clinical or cognitive feature suggesting a different pathology than CBD.

## Introduction

Corticobasal degeneration (CBD) is a progressive neurodegenerative disorder characterized by levodopa-resistant asymmetric akinetic-rigid parkinsonism and limb dystonia, variably associated with cortical features [1]. Clinicopathological studies reported a high rate of misdiagnosis in patients with clinical diagnosis of CBD [2]–[9], so that it has been suggested that patients fulfilling clinical features of CBD should receive the clinical diagnosis of ‘corticobasal syndrome’ (CBS), until *post-mortem* examination unravels the underlying causative pathology [3], [10]–[12]. An increasing amount of studies investigated clinical, neuropsychological and imaging features of patients with CBD-like phenotype searching for distinctive *in vivo* features, with variable and overall not conclusive results [9], [13]–[15].

Striatal dopamine transporter (DAT) imaging is a sensitive biomarker of substantia nigra pars compacta (SNc) neuronal density that may improve the accuracy of *in vivo* diagnosis in patients with CBS, given that SNc cell loss is a pathological finding considered to be mandatory for the definite diagnosis of CBD [3], [16], [17]. Nevertheless, *ante-mortem* evidences of preserved presynaptic nigrostriatal terminals in CBS patients who subsequently developed severe SNc degeneration [18], [19] raise the need to clarify possible delayed ‘timing’ of SNc terminal loss in relation to the emergence of motor symptoms.

In the present study, we investigated *in vivo* DAT density using single photon emission computed tomography (SPECT) in a large cohort of patients with CBS, along with extensive clinical, neuropsychological and brain magnetic resonance imaging (MRI) characterization. Our primary objective was to describe the pattern of DAT reduction in CBS compared to PD and healthy control subjects and to correlate DAT binding with clinical features. In addition, we aimed to assess the prevalence of preserved SNc neuronal density in CBS population and highlight possible differential features between CBS subjects with normal *vs.* pathological SPECT scans.

## Methods

### Subjects

Thirty-six outpatients with clinical diagnosis of ‘probable CBD’ according to current diagnostic criteria [1] were consecutively recruited from three Italian Movement Disorders Clinics (Parkinson Institute of Milan, Department of Neurosciences of Pisa, and Neurological Unit of Versilia Hospital) between 2005 and 2008. We excluded those patients with exposure to typical neuroleptics; those with brain MRI evidence of moderate-to-severe vascular abnormalities, space-occupying lesions or normal pressure hydrocephalus. We did not exclude patients presenting early cognitive impairment [1], as dementia might be a presenting feature of CBD [20]. We recorded the most prominent motor and non-motor symptoms and signs of CBD at neurological examination [1], [5] as well as presenting clinical features according to patients' previous data (clinical signs were considered positive only if they were identified by a neurologist) or, when not available, as reported by the subject or caregiver as significant complaints. We considered as ‘early’ those features that occurred within the first 12 months from the clinical onset.

Motor function assessment in CBS and PD patients included the Hoehn and Yahr stage (H&Y) and the Unified Parkinson Disease Rating Scale motor score (UPDRS III) assessed in the morning while on their current therapy. Twenty-six CBS patients were receiving levodopa, 12 were taking anticholinergics and/or benzodiazepines while none was taking dopamine agonists, COMT or MAO-B inhibitors, amantadine, selective serotonin uptake inhibitors, or cholinesterase inhibitors. In the PD control group, individual levodopa equivalent daily dose (LEDD) at the time of SPECT scan was calculated as the sum of the levodopa and dopamine agonists daily dosage, as reported elsewhere [21]. Dopaminergic medication has been demonstrated not to play a significant influence on FP-CIT uptake [22], [23].

At the time of FP-CIT SPECT imaging, all CBS patients underwent full neuropsychological evaluation using the following scales: the mini mental state examination (MMSE) [24], the Corsi block tapping test, digit span, story recall, frontal assessment battery (FAB) [25], attentional matrices, verbal fluencies using category and phonemic cues (1-minute), and neuropsychiatric inventory (NPI) [26]. We applied adjustment for age and education when appropriate and used pathological cut-off values as established elsewhere [27]–[33]. We additionally recorded the occurrence of dementia according to Diagnostic and Statistical Manual of Mental Disorders Fourth Edition (DSM IV) criteria [34]. As CBS patients were assessed on-medications, we could not exclude a potential confounding effect of anticholinergic medications on cognitive function. We used two control populations to compare FP-CIT SPECT imaging binding values: one consisting of 37 patients with idiopathic PD according to the UK Brain Bank diagnostic criteria [35] of similar age and disease duration and a second group of 32 age-matched healthy control (HC) subjects who had no history or evidence of neurologic or psychiatric disease. All CBS patients underwent 1.5 Tesla brain MRI, performed according to a previously published protocol [36]. MR images were evaluated by an independent neuroradiologist (unaware of the clinical and FP-CIT imaging status of patients) who was asked to record the following features: asymmetric or symmetric frontal and/or parietal cortical atrophy, diffuse cortical atrophy, subcortical atrophy, midbrain atrophy, normal scan.

The study was approved by the Ethics Committees of the institutions involved in the study. Subjects' consent was obtained according to the Declaration of Helsinki.

### FP-CIT SPECT

SPECT studies were carried out either at the Nuclear Medicine, University of Pisa, or at the IRCCS-Ospedale Maggiore, Milan. Intravenous administration of 110–185 MBq of ^123^I-2β-carbometoxy-3β-(4-iodophenyl)-N-(3-fluoropropyl) nortropane (^123^I-FP-CIT) (DaTscan®, GE Healthcare) was performed 30–40 min after thyroid blockade in all subjects after overnight withdrawal of dopaminergic medications. Brain SPECT was performed 3–4 hours later by means of a dedicated triple detector gamma camera (Prism 3000, Philips, Eindhoven, The Netherlands) equipped with ultra-high-resolution fan beam collimators (four subsets of acquisitions) or a dual-head gamma camera (Optima NT, GE Healthcare, Milwaukee, WI, USA) equipped with high resolution low energy parallel holes collimators, matrix size 128×128, radius of rotation 13–15 cm, 120–128 projections over a 360° circular orbit. The overall scanning time for each patient was 30 to 45 minutes. Brain sections were obtained by applying an iterative algorithm (OS-EM, 2–4 iterations and 8–15 subsets) on projection data and a 3D postfilter (Butterworth, order 5, cut-off 0.3 Ny) and attenuation correction (Chang method, μ = 0.12 cm^−1^). Transaxial data were reoriented along the fronto-occipital line. Usually five to eight slices were summed in order to include the basal ganglia. Regular circular regions of interest were drawn on the bilateral total striatum, on the caudate nucleus and the putamen and used to calculate the average striatal-to-nonspecific uptake areas with negligible amount of dopamine receptors, such as occipital lobes. The formula used was as follows: FP-CIT binding = [(mean radioactivity in the striatum)−(mean radioactivity in the occipital cortex)]/(mean radioactivity in the occipital cortex). Asymmetry indices (AIs) of FP-CIT binding were calculated as reported by Sherfler and colleagues [37]. The ‘contralateral’ side in CBS and PD patients was defined as the side opposite to the clinically most affected side. For statistical purposes, in healthy subjects we conventionally referred to the side with lower FP-CIT uptake as the ‘contralateral’. The relationship between FP-CIT binding in the caudate nucleus and the putamen contralateral to the most affected body side was calculated for each individual as follows: Caudate-to-putamen ratio = Caudate binding/Putamen binding. Patients and control subjects were balanced between Milan and Pisa nuclear medicine departments.

### Data analysis

All statistical analyses were carried out using BMDP software package for Windows Release 2009 (BMDP Statistical Software, University of California Press, Release 2009 – Berkeley, Los Angeles, Oxford). FP-CIT SPECT binding data (uptake values, AIs in striatum, caudate nucleus and putamen, and caudate-to-putamen ratio) obtained from CBS patients were compared with those obtained from HC subjects and PD patients. Parametric methods were used for variables after testing for normal distribution (Shapiro-Wilk W statistic [38]). Continuous variables were compared using the Student's *t*-tests or analysis of variance (ANOVA). For multiple comparisons between groups, a Bonferroni correction was applied. The Kruskal-Wallis test and the Mann-Whitney U test were used to compare variables without normal distribution. Frequencies were compared by means of χ^2^ test, with Yates correction for 2×2 tables. In addition, differences in neuropsychological testing scores between the CBS patients with normal vs. pathological FP-CIT uptake were calculated using dichotomous ‘normal’ *vs.* ‘pathologic’ variables, according to normative cut-off values. To discriminate between CBS and HC groups we performed linear discriminant analysis with a forward selection mechanism based on Wilk's lambda as selection criteria for potential predictors, i.e. [123I] FP-CIT mean binding values in the bilateral caudate nucleus and the putamen, the caudate-to-putamen ratio, and the hemispheric asymmetry index of the striatal uptake [37]. An additional discriminant analysis was performed using caudate nuclei and putamina uptake values as potential predictors and excluding indirect variables such as asymmetry indices and caudate-to-putamen ratio. For group membership, the same a priori probability was assumed for all cases. For validation of the model we used a leave-one-out procedure. The cut-off value between two groups was determined as follows: (1) the mean and its confidence interval (CI 95%) of both groups were calculated; and (2) the cut-off value was determined by averaging over the lower bound of the CI of one group and the upper bound of the other group. In CBS and PD groups the relationship between clinical variables (disease duration, the UPDRS motor score, the H&Y stage, neuropsychological testing scores) and FP-CIT binding values in the whole striatum, caudate nucleus and putamen were explored using the Pearson correlation coefficient. Neuropsychological testing scores were correlated with right *vs.* left striatal structures rather than ispilateral *vs.* contralateral to the most affected body side.

## Results

Cohort characteristics in CBS, PD and HC subject groups have been summarized in **Table 1**. Compared to PD control subjects, CBS patients had lower LEDD (p = 0.0001) and worse disease severity (p<0.0001).

**Table 1:** Demographic features and ratings in CBS, PD, and healthy control subjects.

Features	CBS (n = 36)	HC (n = 32)	PD (n = 37)
**M∶F**	16∶20	15∶17	18∶19
**Age at onset**	67.2 (6.9)	-	65.5 (5.5)
**Age at FP-CIT SPECT, y**	71.1 (7.3)	69.8 (5.7)	69.9 (5.3)
**Side of symptoms onset, L∶R**	19∶17	-	16∶21
**Disease duration, years**	3.89 (1.6)	-	4.4 (2.9)
**LEDD, mg**	328.5 (263.4)*	-	538.8 (250.0)
**Hoehn and Yahr stage**	3.1 (0.8)*	-	1.91 (0.7)
**UPDRS part III**	38.8 (12.6)*	-	21.7 (7.8)

Intergroup differences of demographic features were calculated by one-way ANOVA with *post hoc* significance correction (*p<0.05 in CBS vs. PD). Values are mean (SD).

### 1. FP-CIT SPECT findings (Table 2, Figures 1 and 2)

**Figure 1:**
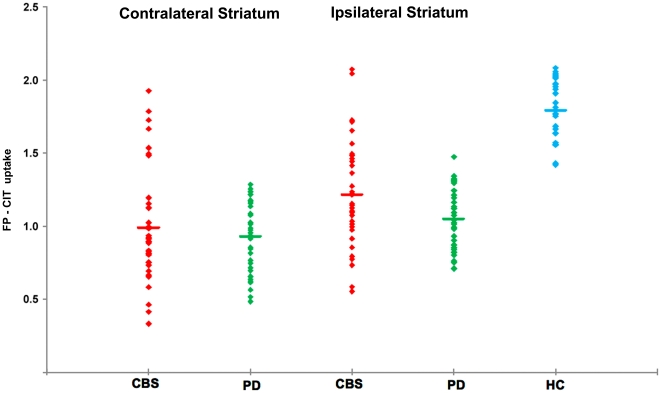
Scatter plot showing individual FP-CIT uptake values in the striatum contralateral and ipsilateral to the most affected body side in CBS, PD, and HC subjects. The uptake values of HC subjects have been averaged between the two sides. The line represents the mean.

**Figure 2:**
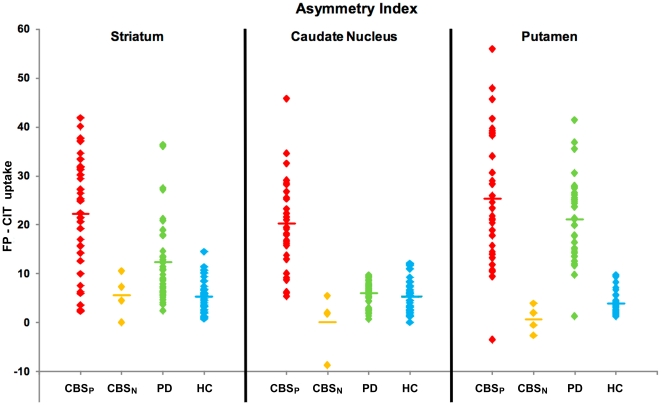
Scatter plots showing hemispheric asymmetry indices in the total striatum, caudate nucleus and putamen in CBS_N_, CBS_P_, PD, and HC subjects.

**Table 2:** Semiquantitative FP-CIT binding values in caudate nucleus and putamen, hemispheric asymmetry indices and caudate-to-putamen ratio in CBS, PD, and healthy control subjects.

FP-CIT SPECT binding values	CBS tot (n = 36)	CBS_N_ (n = 4)	CBS_P_ (n = 32)	HC (n = 32)	PD (n = 37)
**Contralateral caudate**	1.04 (0.4)	1.66 (0.1)	0.96 (0.3)	1.77 (0.2)	1.25 (0.3)
**Ipsilateral caudate**	1.26 (0.4)	1.67 (0.2)	1.21 (0.4)	1.82 (0.2)	1.33 (0.3)
**Contralateral putamen**	0.92 (0.4)	1.63 (0.2)	0.84 (0.4)	1.68 (0.1)	0.68 (0.2)
**Ipsilateral putamen**	1.17 (0.4)	1.64 (0.1)	1.11 (0.4)	1.71 (0.2)	0.87 (0.2)
**Asymmetry index Caudate, %**	17.93 (10.8)	4.45 (3.3)	20.17 (9.1)	5.28 (3.3)	5.98 (2.5)
**Asymmetry index Putamen, %**	22.50 (15.1)	2.27 (1.3)	25.25 (13.6)	3.8 (2.3)	21.14 (8.3)
**Caudate-to-putamen ratio**	1.19 (0.3)	1.02 (0.1)	1.21 (0.3)	1.07 (0.1)	1.88 (0.5)

The whole cohort of CBS patients has been divided into those with normal (CBS_N_) and pathological (CBS_P_) FP-CIT binding. Values are mean (SD).

#### CBS patients vs. PD control subjects

When compared to PD control subjects, CBS patients showed larger variability in the severity and pattern of FP-CIT binding reduction, with more uniform reduction throughout the striatum and greater hemispheric asymmetry. FP-CIT uptake in CBS was similar in the striatum contralateral to the most affected body side and higher ipsilaterally than PD (p<0.001); it was lower in the contralateral caudate nucleus (p = 0.005) and similar ipsilaterally, higher in contralateral and ipsilateral putamen (p<0.001). Caudate AI was significantly higher in CBS than PD (p<0.0001), while putaminal AI was similar. CBS had lower caudate-to-putamen ratio than PD (p<0.0001).

#### CBS patients vs. HC subjects

CBS had reduced FP-CIT uptake and higher AIs in the caudate nucleus and putamen and similar caudate-to-putamen ratio in comparison to HC (p<0.0001 in each comparison). Using linear discriminant analysis, we found that thirty-two patients with CBS had pathological FP-CIT binding (hereafter referred to as ‘CBS_P_’) while four cases had normal FP-CIT binding (hereafter referred to as ‘CBS_N_’; Wilk's Lambda = 0.075, F-Statistic = 42.840). In the CBS_P_ group were also included three cases (age 62±5.5 years; disease duration 3.3±1.1 years; H&Y stage 2.5±0.5) whose FP-CIT binding was in the normal range but whose hemispheric AI was significantly higher than PD control subjects (Wilk's Lambda = 0.1653; cases 5, 6 and 7 in **Table 3**). Other four CBS_P_ subjects had normal striatal uptake ipsilateral to the most affected body side but not contralaterally, despite bilateral extrapyramidal signs.

**Table 3:** Demographic features, clinical signs and ratings, FP-CIT uptake measures and additional neuroimaging investigations in the 4 CBS patients with normal FP-CIT measures.

	Case 1	Case 2	Case 3	Case 4	(Case 5)	(Case 6)	(Case 7)
**Clinical features**							
Gender	F	M	F	M	F	M	F
Age at onset	77	64	74	58	67	62	56
Age at SPECT	81	72	78	66	71	64	60
Disease duration at FP-CIT SPECT	4	8	4	8	4	2	4
Levodopa daily dosage	750	0	0	0	400	300	300
Hoehn and Yahr stage	3	4	2,5	2,5	2,5	2	3
UPDRS III	37	38	25	25	32	22	30
Language dominant hemisphere	L	L	L	L	L	L	L

Clinical presentation	Gait difficulty.Cognitive impairment.	Hand clumsinessAnd jerk tremor, gait difficultyDepression.	Hand clumsiness, rigidity and tremor. Episodes of Disorientation/confusion.	Hand clumsiness and rigidity.	Hand clumsiness, gait difficulty.	Language production and behavioral disturbances.Hand clumsiness.	Hand clumsiness, gait difficulty, bradykinesia.
**Motor signs**							
Side of motor symptoms onset	R	L	L	R	L	L	L
Akinesia	(+)	(+)	(+)	(+)	(+)	(+)	(+)
Rigidity	(+)	+	(+)	(+)	(+)	(+)	(+)
Tremor	−	(+)	(+)	+	−	+	(+)
Limb dystonia/Clenched fist	+/+	+/+	(+)/+	+/+	+/−	−/−	+/−
Myoclonus	−	+	+	+	−	+	(+)
Gait disturbance	(+)	(+)	−	−	(+)	−	(+)
Postural instability	−	−	−	−	−	−	+
**Cortical/cognitive features**							
Dementia	(+)	+	(+)	−	−	+	+
Limb apraxia	+	(+)	(+)	+	(+)	+	+
Alien limb phenomenon	+	+	−	+	+	+	+
Cortical sensory deficit	+	*+*	*+*	*+*	−	−	−
Non-fluent aphasia	+	+	+	+	−	(+)	(+)
Early memory impairment	−	−	+	−	−	−	+
Early visuo-spatial dysfunction	+	+	+	−	−	+	+
Behavioral abnormalities/personality changes	−	−	−	−	−	(+)	+
**Other features**							
Dysarthria and/or Dysphagia	−	+	−	+	+	−	−
Oculomotor impairment	−	+	−	−	+	+	−
Depression	(+)	(+)	−	+	(+)	+	(+)
Frontal-lobe release signs	+	+	+	−	+	+	+

Other frontal/parietal lobe signs	Gait apraxia	Left-sided neglect, gait apraxia	Left-sided neglect, prosopoagnosis.	Right-sided Neglect	-	-	Gait apraxia
**Neuropsychological testing**							
MMSE	16	20	13	28.9	23.7	17.9	18
Corsi Block Tapping Test	2.5	2	2	4	4.75	2.25	2.11
Digit Span	4	4.4	5	4.75	3.75	3.5	3.5
Story recall	7.5	5	4.3	8.2	13.8	8.8	n.a.
Attentional Matrices	13.5	15	26	33	31.5	11.5	n.a.
Category Verbal Fluency	n.a.	17	14	24	n.a.	n.a.	n.a.
Phonemic Verbal Fluency	8	13	18	14	13	10.9	7.2
Frontal Assessment Battery	6	7	9	12.9	11.8	10	5
Neuropsychiatric Inventory	20	15	12	14	6	16	18
**FP-CIT SPECT binding**							
Contralateral striatum	1,78	1,53	1,49	1,48	1,72	1,66	1,92
Ipsilateral striatum	1,65	1,71	1,56	1,48	2,07	1,72	2,04
Contralateral caudate	1,60	1,77	1,72	1,54	1,73	1,50	2,08
Ipsilateral caudate	1,47	1,87	1,75	1,57	2,36	1,78	2,48
Contralaterale putamen	1,87	1,51	1,57	1,58	1,65	1,74	1,84
Ipsilaterale putamen	1,82	1,57	1,60	1,57	1,90	1,68	2,06
Asymmetry index (Striatum)	7,30	10,53	4,49	0,00	16,91	3,49	5,88
Asymmetry index (Caudate nucleus)	−8,84	5,35	1,71	1,91	26,69	15,73	16,13
Asymmetry index (Putamen)	−2,75	3,82	1,88	−0,64	13,16	−3,57	10,68
Caudate∶Putamen ratio	0,86	1,17	1,10	0,97	1,05	0,86	1,13

We additionally reported 3 CBS cases with normal FP-CIT uptake in the caudate nucleus and the putamen bilaterally, but included in the CBS_P_ subgroup because of abnormal asymmetry indices and/or caudate-to-putamen ratio (cases 5, 6 and 7). + = sign present; (+) = initial sign; − = sign absent.

#### CBS subgroups analysis: CBS_N_ vs. CBS_P_

FP-CIT binding values were significantly higher in the bilateral caudate nucleus and putamen of CBS_N_ compared to CBS_P_ (p<0.001 in each comparison) and similar to HC subjects. Kruskall-Wallis test found significant differences on the mean ranks of caudate nucleus and putaminal FP-CIT uptake among CBS_N_, CBS_P_, PD, HC (p<0.0001). *Post-hoc* analysis showed that FP-CIT uptake in the caudate nucleus of CBS_N_ was similar to HC and PD, while putaminal uptake was higher than PD (p = 0.006) and similar to HC, bilaterally. Striatal uptake in CBS_P_ was similar to PD and lower than HC (p<0.0001). CBS_P_ patients had higher caudate and putamen AIs compared to CBS_N_ and HC (p<0.0001 in each comparison), while they showed only higher caudate AI (p<0.0001) compared to PD. CBS_N_ had similar caudate and putaminal AIs compared to HC, while they had similar caudate and lower putaminal AI (p = 0.0001) compared to PD. The mean caudate-to-putamen ratio was similar in CBS_N_ vs. CSB_P_ vs. HC, while this was higher in PD than all the other groups (p<0.001 in each comparison).

### 2. Clinical, neuropsychological and brain MRI features

**Figure 3:**
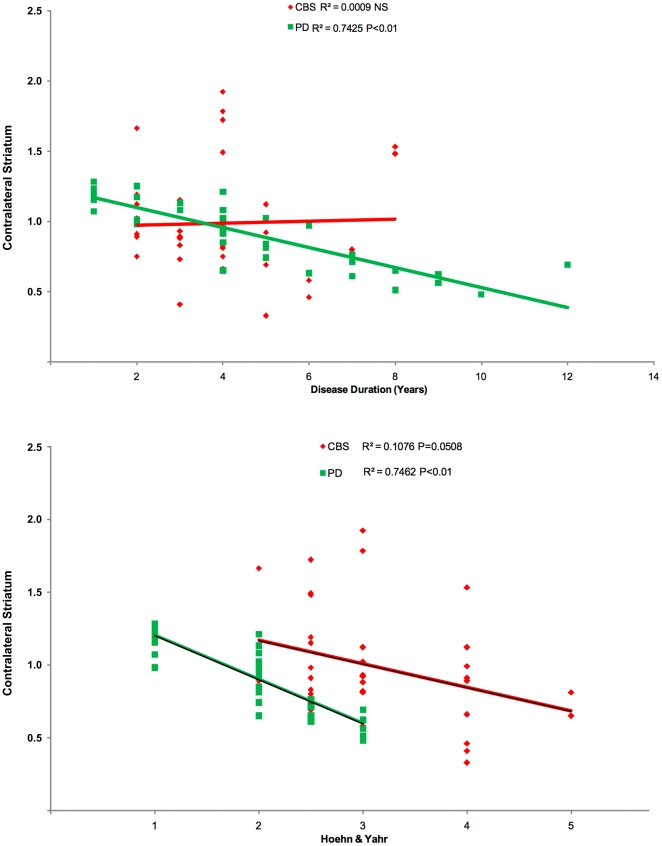
Correlation of FP-CIT binding in the contralateral striatum. Shown with the disease duration (A), and disease severity, according to the H&Y stage (B), in CBS patients and in PD controls.

In the whole group of CBS patients (n = 36), extrapyramidal signs were similarly recorded in 94% of cases in the early stages of the disease, irrespective of FP-CIT uptake. Patients presented with either akinetic (92%), dystonic (11%), apraxic (67%) arm, or gait disorder/postural instability (30%) (**Table S1**). During the disease course, all patients developed full-blown typical clinical picture of CBS and none experienced significant and sustained clinical improvement from chronic levodopa treatment. Disease duration was negatively correlated with FP-CIT uptake in the striatum, the caudate nucleus and the putamen only in PD (p<0.01 in all the analyses) but not in CBS (**Figure 3A**). This difference remained significant also after excluding CBS_N_ subjects from the analysis (data not shown). H&Y stage negatively correlated with striatal FP-CIT binding in the PD group (p<0.01) while showed a trend for negative correlation with striatal binding in CBS patients (**Figure 3B**). The UPDRS motor score showed negative correlation with striatal uptake in PD (p<0.01) and CBS (p = 0.047) groups. Neuropsychological profile of the whole CBS cohort was overall characterized by mildly reduced global cognitive performance, characterized by reduced frontal lobe functions and relatively preserved verbal and visuospatial short- and long-term memory tasks (**Table S2**). We did not find any correlation between FP-CIT binding and any of the neuropsychological scores.

We applied non-parametric tests to investigate the differences between CBS_N_ and CBS_P_ (**Table S1**). Demographic and general clinical features were similar between the two CBS subgroups, with the exception of mean longer disease duration in CBS_N_ at the time of SPECT (p = 0.038). Clinical presentation did not significantly differ between the two CBS subgroups, with the only exception of early memory impairment, that was more frequently reported in the clinical history of CBS_N_ patients (p<0.005). At the time of SPECT scan, CBS_P_ patients had higher prevalence of postural instability and falls than CBS_N_ (p = 0.005) while parietal lobe signs other than apraxia were more frequently reported in CBS_N_ *vs.* CBS_P_ (p<0.001). Neuropsychological testing scores did not differ between CBS_N_ and CBS_P_ (**Table S2**). Brain MRI findings were not different between CBS_N_ *vs.* CBS_P_ (**Table S3**). Finally, statistical analyses performed lumping together all CBS patients showing normal FP-CIT uptake values (n = 7 subjects, **Table 3**) confirmed the lack of any significant difference between CBS_N_ vs. CBS_P_ in all the clinical, neuropsychological and MRI variables investigated, including disease duration (4.9±2.3 *vs.* 3.7±1.4, p = 0.08).

## Discussion

This is the first study investigating presynaptic dopaminergic function along with extensive clinical and neuropsychological characterization in a large cohort of patients fulfilling clinical criteria for ‘probable CBD’. The primary outcome of this study was to describe the pattern of striatal FP-CIT uptake reduction in CBS and correlate FP-CIT binding with clinical features. Compared to PD control subjects, individuals with CBS showed a large FP-CIT binding variability in terms of overall striatal binding as well as hemispheric asymmetry and caudate-to-putamen ratio (**Figures 1** and **2**). In consistence with typical CBD pathological features [17], we found higher hemispheric asymmetry in caudate nucleus and putaminal FP-CIT uptake together with a more uniform reduction within striatal regions in CBS, thus reflecting the highly asymmetrical involvement and SNc neuronal loss involving both dorsal and ventral tiers in CBD [16], [17] in contrast to PD, where cell loss is typically confined to SNc ventral tiers [39]. These features well explain the difference in the caudate-to-putamen ratio of FP-CIT uptake between the two disorders. When correlation analysis was performed between nigrostriatal function and clinical features in CBS, FP-CIT binding showed did not show any correlation with disease duration and just a trend with disease severity, while this association was highly significant in PD control subjects (**Figure 3A and B**). Previous imaging studies already highlighted a less pronounced SNc neuronal loss in patients with CBS compared not only to severe atypical parkinsonian syndromes (such as progressive supranuclear palsy and multiple system atrophy) but also to idiopathic PD [40]–[44]. Interestingly, some CBS patients had symmetrical FP-CIT reduction despite strongly asymmetrical symptoms, a finding already described in a previous fluorodopa-PET study [41]. A recent description of autopsy-proven CBD with *ante-mortem* evidence of preserved presynaptic nigrostriatal terminals suggested a possible delayed timing of SNc cell degeneration, unrelated to the onset of motor symptoms [18], [19]. The lack of relationship between FP-CIT binding and disease duration and the very weak correlation with disease severity we found in our CBS cohort is in line with this hypothesis. Moreover, the presence of obvious bilateral symptoms but evidence of reduced DAT density only unilaterally in some CBS cases would further suggest that SNc pathology may require a variable time to occur bilaterally. These findings highlight a mismatch between SNc neuronal loss and clinical features (such as asymmetry of motor symptoms, disease duration and severity) and support the hypothesis of a prominent role played by cortical and/or striatal pathology in the clinical picture of this syndrome. The lack of any correlation between FP-CIT binding and cognitive performance is an expected finding that is consistent with ‘cortical’ rather than ‘subcortical’ pathology underlying cognitive deficits in CBS compared to PD.

As secondary objective, we aimed to assess the prevalence and characterize those CBS subjects with normal FP-CIT scans aiming to find any distinctive clinical or neuropsychological feature. To achieve this objective, we used very restrictive *a priori* criteria to minimize false positive cases and found preserved SNc neuronal density in approximately 10% of our cases. So far, normal dopamine transporter density has been reported only sporadically in CBS [19], [45], while none of the few systematic imaging studies in consecutive cohorts ever documented any normal scan [40]–. To our knowledge, there are only few reports of patients with clinical diagnosis of CBD with preserved SNc neuronal density at *post-mortem*, and most of them displayed either Alzheimer's or Pick's disease pathology [2], [3], [5], [7], [9], [10], [14], [15], [46]–[48]. The first possible explanation would come from a possible pathological involvement of the downstream postsynaptic neurons, even though the pathological involvement of postsynaptic striatal neurons in CBD is variable and unpredictable [17], [49]. A second hypothesis would posit that extrapyramidal symptoms in CBS with normal SNc density might be associated with frontal and/or parietal cortical rather than striatal pathology [4], [7], [19], [50], [51]. In the attempt to rule this out, we extensively investigated clinical, neuropsychological and MR imaging features of our CBS cohort and did not find any major clue suggesting an alternative diagnosis than CBD. Although early memory impairment was more frequently recorded in the history of CBS_N_ cases (**Table S1**), full neuropsychological assessment revealed similar features in both subgroups, including preserved memory functions, consistent with the pattern shown in definite CBD (**Table S2**) [52]. Notably, our cohort is representative of specialized movement disorders centres so that the number of cases presenting with prominent cognitive impairment and mild-to-absent extrapyramidal symptoms is relatively low, as these subjects would rather refer to third-level dementia clinics [20], [52]. SNc depigmentation is less evident in CBD cases presenting with severe cognitive symptoms [53] and it is thus conceivable that we underestimated the real prevalence of normal FP-CIT scans in CBS population [6], [20]. Moreover, CBS subjects with normal scans showed a mean longer disease duration than CBS patients with presynaptic nigrostriatal degeneration despite similar degrees of motor disability, suggesting a more indolent course.

We acknowledge that the interpretation of our findings is limited by the lack of pathological confirmation of the diagnosis. Indeed, some CBS patients with normal FP-CIT binding might still turn out to be focal cortical variants of other dementia than CBD [3], [7], [10], [46], [47], [54]–[59], because clinical features and the distribution of pathological lesions are closely associated whatever the histologic nature [3], [4], [9], [14]. *Post-mortem* assessment in CBS cases with preserved SNc neuronal density could also unravel the pathological underpinnings of extrapyramidal symptoms. A second limitation in the interpretation of the comparison between CBS_N_ and CBS_P_ comes from the marked unbalance in the number of cases between the two groups.

Taken as a whole, our findings support the hypothesis of a mismatch between SNc neuronal loss and clinical features with a variable contribution played by supranigral pathology in extrapyramidal phenotype. We speculate that the well-known heterogeneity of CBD pathology [60] might additionally include variants with predominant involvement of the cerebral cortex and disease progression in the rostro-caudal direction to the brainstem, in a kind of ‘*cortico-to-basal*’ progression of neuronal degeneration. This hypothesis is supported by pathological studies showing a less evident SNc depigmentation in CBD cases presenting with cognitive rather than motor symptoms [53] and already demonstrated in Lewy bodies dementia [61], [62]. Clinicopathological studies including *in vivo* DAT imaging are needed to assess the predictive value of normal presynaptic nigrostriatal function in the diagnostic work up of patients with CBD-like phenotype.

### Conclusions

*In vivo* assessment of dopamine transporter SPECT imaging in a large CBS population was found to be normal in about 10% of cases despite prominent bilateral extrapyramidal signs. In these cases, clinical and neuropsychological features were not distinct from those with evidence of SNc neuronal loss. The lack of any correlation between presynaptic nigrostriatal dysfunction and disease duration might suggest an unpredictable and possibly delayed SNc degeneration in CBD and further supports the hypothesis of a variable contribution of supranigral pathology to its motor phenotype.

## Supporting Information

Table S1 **Clinical signs of CBS_N_ and CBS_P_ at the time of FP-CIT SPECT.** Features are reported as number of patients (%) within each subgroup. Extrapyramidal signs refer to the combination of bradykinesia *and* rigidity. Yates corrected χ^2^ test was applied, ^*^p<0.005.
(DOC)

Table S2 **Neuropsychological testing of CBS patients with normal (CBS_N_) and pathological (CBS_P_) FP-CIT uptake.** Values have been adjusted for age and education and given as mean (SD).
(DOC)

Table S3 Brain MRI features of all CBS patients (CSBtot) and the two subgroups of those with normal (CBS_N_) and pathological (CBS_P_) FP-CIT uptake values. Features are reported as N° of patients (%). Intergroup differences were calculated by Yates corrected χ^2^ test.
(DOC)
