# Supplementary material for: Dopamine Transporter SPECT Imaging in Corticobasal Syndrome
Source: PLoS One. 2011 May 2;6(5):e18301. doi: 10.1371/journal.pone.0018301 (PMC3085517; doi:10.1371/journal.pone.0018301)
Supplement: Table S1 — Clinical signs of CBSN and CBSP at the time of FP-CIT SPECT. Features are reported as number of patients (%) within each subgroup. Extrapyramidal signs refer to the combination of bradykinesia and rigidity. Yates corrected χ2 test was applied, *p<0.005. (DOC) [file pone.0018301.s001.doc]

**Supplementary Table S1**

| **Feature** | **CBStot, n=36** | **CBS_N_, n=4** | **CBS_P_, n=32** |
| --- | --- | --- | --- |
| **Motor signs** |  |  |  |
| Extrapyramidal signs | 34 (94.4) | 4 (100) | 30 (94) |
| Early bradikinesia | 33 (91.7) | 4 (100) | 29 (91) |
| Early rigidity | 21 (58.3) | 3 (75) | 18 (56) |
| Tremor | 18 (50) | 3 (75) | 15 (47) |
| Early tremor | 11 (30.5) | 2 (50) | 9 (28) |
| Limb dystonia | 27 (75) | 4 (100) | 23 (72) |
| Early limb dystonia | 4 (11.1) | 1 (25) | 3 (9.4) |
| Myoclonus | 23 (63.9) | 3 (75) | 20 (57) |
| Postural instability | 24 (66.7) | 0 (0) | 24 (75) |
| Early postural instability | 11 (30.5) | 0 (0) | 11 (34.4) |
| **Cortical/cognitive signs** |  |  |  |
| Dementia | 16 (44.4) | 3 (75) | 13 (41) |
| Limb apraxia | 36 (100) | 4 (100) | 32 (100) |
| Early limb apraxia | 24 (66.7) | 2 (50) | 22 (69) |
| Alien limb phenomenon | 17 (47.2) | 3 (75) | 14 (44) |
| Cortical sensory loss / Hemisensory neglect | 19 (52.7) | 4 (100) | 15 (47) |
| Aphasia | 16 (44.4) | 3 (75) | 13 (41) |
| Early speech difficulties | 8 (22.2) | 0 (0) | 8 (25) |
| Early memory impairment | 10 (27.8) | 4 (100)* | 6 (19) |
| Early visuo-spatial dysfunction | 15 (41.7) | 1 (25) | 14 (44) |
| Early behavioral / Personality abnormalities | 14 (38.9) | 1 (25) | 13 (41) |
| **Other clinical features** |  |  |  |
| Oculomotor impairment | 17 (47.2) | 2 (50) | 15 (47) |
| Dysarthria and/or dysphagia | 22 (61.1) | 1 (25) | 21 (66) |
| Early dysarthria and/or dysphagia | 5 (13.9) | 0 (0) | 5 (16) |
| Frontal-lobe release signs | 33 (91.7) | 3 (75) | 30 (94) |
