# Supplementary material for: Dopamine Transporter SPECT Imaging in Corticobasal Syndrome
Source: PLoS One. 2011 May 2;6(5):e18301. doi: 10.1371/journal.pone.0018301 (PMC3085517; doi:10.1371/journal.pone.0018301)
Supplement: Table S2 — Neuropsychological testing of CBS patients with normal (CBSN) and pathological (CBSP) FP-CIT uptake. Values have been adjusted for age and education and given as mean (SD). (DOC) [file pone.0018301.s002.doc]

**Supplementary Table S2**

| **Neuropsycological testing** | **CBStot** | **CBS_N_, n=4** | **CBS_P_, n=32** | **Cut-off values** |
| --- | --- | --- | --- | --- |
| **MMSE** | 22.7 (5.0) | 19.5 (6.9) | 23.1 (4.7) | 24 |
| **Frontal Assessment Battery** | 9.6 (3.7) | 8.7 (3.0) | 9.7 (3.9) | 13.4 |
| **Attentional Matrices** | 27.1 (11.3) | 21.9 (9.3) | 27.8 (11.4) | 30 |
| **Digit Span** | 4.1 (1.0) | 4.5 (0.4) | 4.1 (1.1) | 3.5 |
| **Story recall** | 8.1 (3.6) | 6.2 (1.9) | 8.4 (3.7) | 4.5 |
| **Corsi Block Tapping Test** | 3.3 (1.0) | 2.6 (0.9) | 3.4 (1.0) | 3.5 |
| **Category Verbal Fluency** | 20.8 (9.3) | 18.3 (5.1) | 21.1 (9.8) | 24 |
| **Phonemic Verbal Fluency** | 14.9 (7.2) | 13.2 (4.1) | 15.1 (7.5) | 16 |
| **Neuropsychiatric Inventory** | 12.7 (8.2) | 15.2 (3.4) | 12.3 (8.7) | n.a. |
