# Supplementary material for: Dopamine Transporter SPECT Imaging in Corticobasal Syndrome
Source: PLoS One. 2011 May 2;6(5):e18301. doi: 10.1371/journal.pone.0018301 (PMC3085517; doi:10.1371/journal.pone.0018301)
Supplement: Table S3 — Brain MRI features of all CBS patients (CSBtot) and the two subgroups of those with normal (CBSN) and pathological (CBSP) FP-CIT uptake values. Features are reported as N° of patients (%). Intergroup differences were calculated by Yates corrected χ2 test. (DOC) [file pone.0018301.s003.doc]

**Table S3.** Brain MRI features of all CBS patients (CSBtot) and the two subgroups of those with normal (CBS_N_ ) and pathologic (CBS_P_) FP-CIT uptake values. Features are reported as N^o^ of patients (%). Intergroup differences were calculated by Yates corrected χ^2^ test.

| **Brain MRI** | **CBStot, n=36** | **CBS_N_, n=4** | **CBS_P_, n=32** |
| --- | --- | --- | --- |
|  |  |  |  |
| Cortical |  |  |  |
| Asymm frontal and/or parietal | 25 (69.4) | 4 (100) | 21 (66) |
| Symmetric frontal and/or parietal | 3 (8.3) | 0 (0) | 3 (9) |
| Diffuse cortical atrophy | 8 (22.2) | 0 (0) | 8 (25) |
| Normal scan | 3 (8.3) | 0 (0) | 3 (9) |
| Subcortical |  |  |  |
| Subcortical atrophy | 21(58.3) | 4 (100) | 17 (53) |
| Midbrain atrophy | 6 (16.7) | 1 (25) | 5 (16) |
